# Depression and Dementia in Old-Old Population: History of Depression May Be Associated with Dementia Onset. The Tome Project

**DOI:** 10.3389/fnagi.2017.00335

**Published:** 2017-10-17

**Authors:** Yi-Chien Liu, Kenichi Meguro, Kei Nakamura, Kyoko Akanuma, Masahiro Nakatsuka, Takashi Seki, Shutaro Nakaaki, Masaru Mimura, Norito Kawakami

**Affiliations:** ^1^Division of Geriatric Behavioral Neurology, Cyclotron and Radioisotope Center (CYRIC), Tohoku University, Sendai, Japan; ^2^Neurological Center of Cardinal Tien Hospital, Taipei, Taiwan; ^3^Fu Jen University School of Medicine, Taipei, Taiwan; ^4^Department of Neuropsychiatry, Keio University, Tokyo, Japan; ^5^School of Public Health, University of Tokyo, Tokyo, Japan

**Keywords:** senile depression, history of depression, dementia, CIDI, MRI

## Abstract

**Background:** In this study, we investigated the relationship among a history of depression, depressive states, and dementia in a community-based old-old cohort.

**Methods:** From 2012 to 2013, we recruited 200 subjects residing in Tome, Japan. Ultimately, 181 subjects were enrolled in our study and completed the whole study protocol. We used the World Mental Health-Composite International Diagnostic Interview 3.0 to evaluate whether subjects had a history of depression or other affective disorders. Simultaneously, 3.0 Tesla brain magnetic resonance imaging (MRI) was performed for each subject.

**Results:** Of 181 subjects, 66 were normal (clinical dementia rating [CDR] = 0), 88 had MCI (CDR = 0.5), and 27 had dementia (CDR = 1 or above). Nine of the 181 subjects (4.9%) had a history of depressive episodes. CDR was significantly higher in subjects with a history of depression (0.9 vs. 0.4, *p* = 0.046) than in those without it. Seventy-two of the 181 subjects (39.7%) exhibited depressive symptoms. Subjects with depression exhibited lower Mini–Mental State Examination scores (21.6 vs. 23.3, *p* = 0.008), higher CDR scores (0.6 vs. 0.3, *p* = 0.004), and more atrophy of the medial temporal lobe (4.4 vs. 3.7, *p* = 0.036).

**Conclusion:** A history of depression should be considered a risk factor for all-cause dementia. In the old-old population, depression is associated with a higher prevalence of dementia, lower cognitive performance, and a smaller hippocampus.

## Introduction

Depression and dementia are very common mental disorders in the elderly population. However, their relationship is not yet fully understood. A systemic review of Alzheimer disease found that a history of depression is an important risk factor (Ownby et al., 2006). Other studies have also demonstrated a strong relationship between late-life depression and all-cause dementia (Gatz et al., 2005; Li et al., 2011; Diniz et al., 2013). Although depression is a common symptom in early stage of Alzheimer's disease (Spalletta et al., 2010), whether depressive symptom persisted or recovered during follow up is also an important predictor of further cognitive deterioration (Spalletta et al., 2012). However, inconsistent findings have been obtained for the relationship between depression and dementia. Several community-based longitudinal studies have not found a connection between depression and dementia development (Becker et al., 2009; Luppa et al., 2013). One twin-based study even found depression to be a prodrome of dementia, but early-life depression was not identified as a risk factor (Brommelhoff et al., 2009).

Several hypotheses have been proposed to explain the possible connection between depression and dementia. The most well-known and consistent hypothesis is the presence of white matter hyperintensities (WMHs) on magnetic resonance imaging (MRI) (Herrmann et al., 2007). WMHs may disrupt the connecting fibers between cortical and subcortical areas in the brain. Most studies have found that WMHs possibly contribute to the pathogenesis of both late-life depression and Alzheimer disease (O'Brien et al., 1996; Taylor et al., 2003). Deep white matter lesions may also be more related to late-life depression. By contrast, periventricular lesions may be more related to Alzheimer disease (Krishnan et al., 2006). In addition to their relationship with depression, WMHs may even impair attention and executive functions in subjects without dementia (Ishikawa et al., 2012). Some studies have demonstrated that subjects with WMHs exhibit poor prognosis and poor responses to medication (Godin et al., 2008).

Another possible structural change related to both depression and dementia is a smaller hippocampus, which has been documented in many epidemiological studies (Campbell et al., 2004; Videbech and Ravnkilde, 2004). Reduced hippocampal volume has also been observed in subjects with post-traumatic stress disorder and other psychiatric disorders (Geuze et al., 2004). The chronic effects of stress hormones, including glucocorticoid and catecholamine, have been most frequently implicated as the causative factor of this structural change (Lupien et al., 2007).

In this study, we focused on the old-old population (age more than 75 years) in a community-based sample. In the old-old population, depression is a serious health problem and is related to poor nutrition, a higher institutionalization rate, and a higher mortality rate (Van Leeuwen Williams et al., 2009). However, few studies have focused on depression in this population. In this study, we investigated the relationship among a history of depression, depressive symptoms, and dementia in this population. Especially, we focused on lifetime history of depressive episodes and their relationship between cognitive function. For detailed records of lifetime depressive episodes, we used WMH-CIDI (World Mental Health-Composite International Diagnostic Interview) for diagnostic interview. We also examined any possible white matter changes and hippocampal atrophy in brain MRI by using different visual rating scales.

## Materials and methods

### Participants

This community-based study of depression and dementia, the Tome Project, was conducted in Tome, Miyagi Prefecture, Northern Japan. From 2012 to 2013, we randomly selected 200 residents aged more than 75 years, of whom 181 agreed to participate in our project and completed the study protocol. The study was approved by the Ethics Committee of Tohoku University Graduate School of Medicine. All participants provided written informed consent in accordance with the Declaration of Helsinki.

### Neuropsychological tests

#### Mini–mental state examination

In this study, the Japanese version of the Mini–Mental State Examination (MMSE) (Ideno et al., 2012) was used to evaluate general objective cognitive function.

#### Clinical dementia rating

The clinical dementia rating (CDR) of each participant was determined by the clinical team, comprising medical doctors and public health nurses, who were blinded to the cognitive test results. Before participants were interviewed by the doctors, the public health nurses visited the participants' homes to evaluate their daily activities. The public health nurses completed a semi-structured questionnaire by using the families' observations of the participants' lives. Participants who lived alone were excluded from this investigation. Participants were then interviewed by the doctors to assess their episodic memory, orientation, and other variables. Finally, based on the information provided by the families, the participants' CDR stages were determined at a joint meeting. A reliable Japanese version of the CDR worksheet (Meguro, 2004) was established, and dementia was diagnosed based on the criteria of Diagnostic and Statistical Manual of Mental Disorders, Edition IV (DSM-IV). One author (K.M.) was certified as a CDR rater at the Washington University School of Medicine Alzheimer's Disease Research Center Memory and Aging Project. We used CDR to evaluate the impairment in daily life caused by cognitive disturbance. The evaluation of CDR was based on five levels (0, 0.5, 1, 2, and 3) in six domains (memory, orientation, judgment, problem solving, community affairs, home and hobbies, and personal care).

#### World mental health-composite international diagnostic interview

The World Mental Health-Composite International Diagnostic Interview 3.0 (WMH-CIDI 3.0) is a fully validated, structured diagnostic tool (Kessler and Ustün, 2004). In this study, trained interviewers (a clinical psychiatrist and one of the authors, K.N.) clinically diagnosed recent and lifetime mental disorders by using the Japanese version of the CIDI. A previous study demonstrated good concordance between the clinical diagnosis and the diagnosis obtained using the Japanese version of the WMH-CIDI (Sakai et al., 2003). Diagnostic interviews were conducted to record depressive episodes, including lifetime major depressive disorder (MDD), lifetime major depressive episodes (MDEs), 12-month MDEs, 30-day MDEs, and 30-day MDD with hierarchy.

#### Geriatric depression scale

In this study, we used the Japanese version of the 15-item geriatric depression scale (GDS) to evaluate depressive symptoms in our subjects. This version of the GDS has been validated in the Japanese population (Sakai et al., 2003). All items in the GDS are rated by self-report as 0 or 1, where 0 = yes and 1 = no. Finally, the total score is calculated, with higher scores indicating more severe depression. Depression was defined as scores higher than the cutoff points (4/5). Subjects with scores of 5 or more were considered depressive, and those with scores of 4 or less were considered nondepressive.

### Brain MRI acquisition

All participants received whole-brain MRI scans (Vantage Titan 3T, Toshiba, Tokyo, Japan) in the clinical assessment. 2D axial fluid-attenuated inversion recovery (FLAIR) images (TR/TE = 10,000/108 ms, Inversion time = 2,700 ms, NEX = 1, Voxel size = 0.3125 × 0.3125 × 5 mm^3^), and high-resolution sagittal T1-weighted images (TR/TE = 13.5/5.5 ms, NEX = 1, Voxel size = 0.3618 × 0.3618 × 1 mm^3^) were acquired. The image analysis included a visual rating of WMH on FLAIR and MTA on theT1-weighted images.

#### Evaluation of medial temporal lobe atrophy

**Figure 1:**
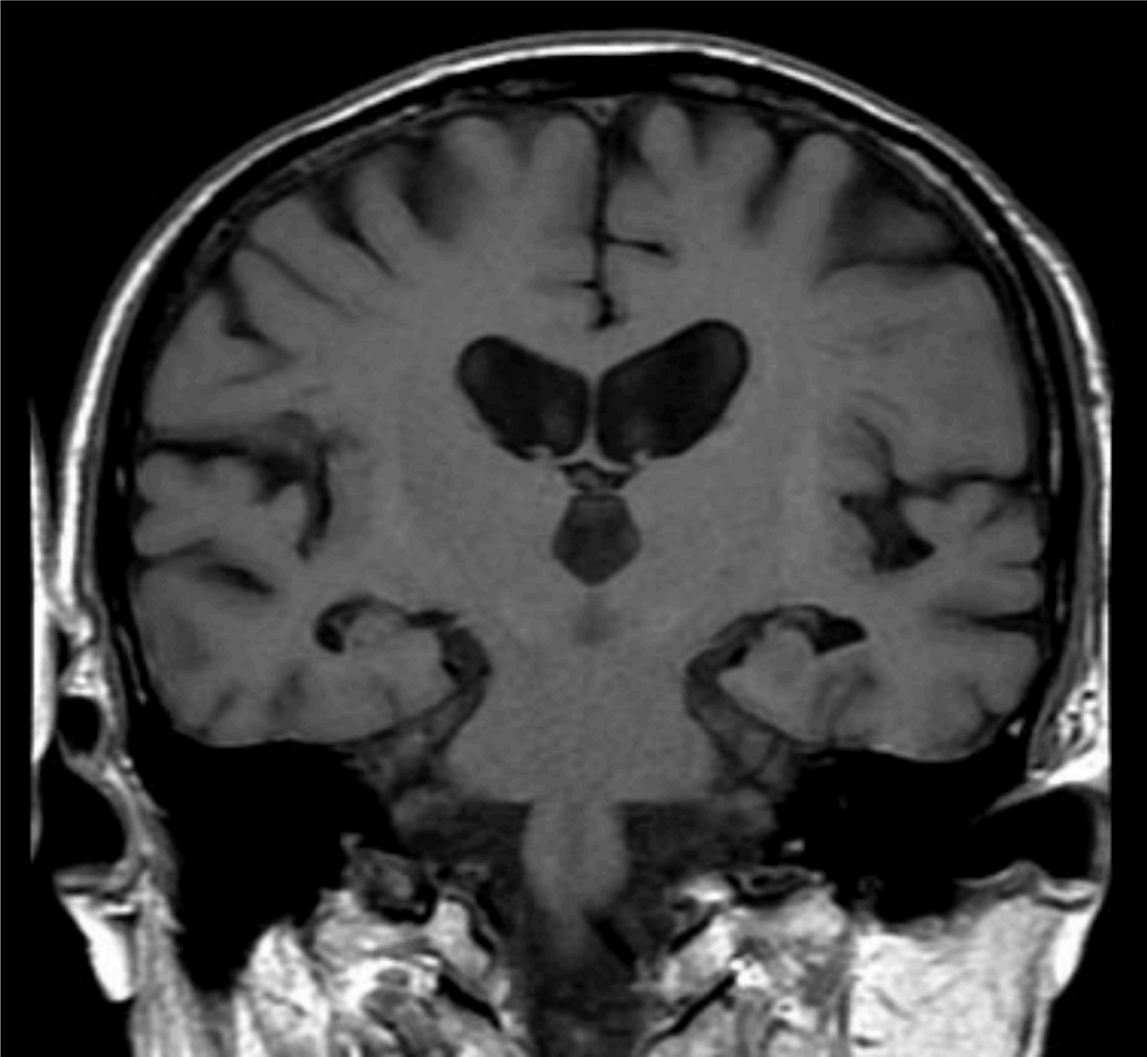
Medial temporal atrophy (MTA) scoring illustrated on T1-weighted MRI. The degree of MTA was rated on a 5-point scale from absent (0) to severe (4). The MTA score of this patient is 2 (right: 2, left: 2).

We evaluated medial temporal lobe atrophy (MTA) in the coronal plane through T1-weighted images. MTA was rated on a 5-point scale (0, absent; 1, minimal; 2, mild; 3, moderate; and 4, severe) based on the height of the hippocampal formation and the width of the choroid fissure and the temporal horn (Kim et al., 2014; Figure 1). The MTA scale was applied to the right and left medial temporal lobe. In our analysis, the dichotomized score of left and right was used.

#### Evaluation of white matter hyperintensities, periventricular hyperintensities, and deep white matter hyperintensities

Lesions adjacent to the lateral ventricles on T2-weighted images were evaluated. The anterior and posterior parts of the periventricular hyperintensities (PVHs) were scored on a 4-point scale (0: absent, 1: minimal, 2: mild, punctuate but thin, 3: definite, and 4: confluent). Finally, the scores of both parts were summed up to yield the total score for PVH. Similarly, deep white matter hyperintensities (DWMHs) were also evaluated on a 4-point scale (0: absent, 1: numbers were less than 5 or the maximum size was <4 mm, 2: numbers were more than 5 and less than 10 or the maximum size was 4–8 mm, 3: numbers were more than 10 and maximum size was >8 mm, and 4: confluent). The scores of bilateral DWMH were finally summed up to yield the total score for DWMH (Ishii et al., 2006).

### Evaluation of cholinergic pathways hyperintensities scale scores

**Figure 2:**
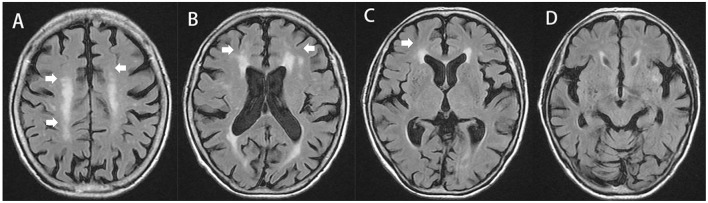
Cholinergic pathways hyperintensities scale (CHIPS) scoring illustrated on T2-FLAIR MRI. The total score of this subject is 12. **(A)** Central semiovale: anterior (right = 1, left = 1, factor = 1, and total = 2); posterior (right = 2, left = 1, factor = 1, and total = 3), **(B)** Corona radiata: anterior (right = 1, left = 1, factor = 2, and total = 4); posterior (right = 0, left = 0, and total = 0); cingulate (right = 0, left = 0, and total = 0), **(C)** High external capsule: anterior (right = 1, left = 0, factor = 3, and total = 3), posterior (right = 0, left = 0, and total = 0); cingulate (right = 0, left = 0, and total = 0), **(D)** Low external capsule: anterior (right = 0, left = 0, and total = 0), posterior (right = 0, left = 0, and total = 0). White arrows highlights the abnormalities scored by CHIPS.

Using FLAIR images, we evaluated WMH lesions involving the cholinergic pathways. The visual rating of the cholinergic pathways hyperintensities scale (CHIPS) required four representative axial images. These four index images included the medial (cingulate gyrus) and lateral (external capsule, claustrum) cholinergic pathways. Using landmarks of the third ventricle, lateral ventricle, and corpus callosum, we analyzed 10 regions using a 3-point scale (0: normal, 1: mild, and 2: moderate to severe) (Figure 2). Each slice was weighed to account for the decreasing concentration of cholinergic fibers. Finally, the total CHIPS score ranged from 0 to 100 (Bocti et al., 2005).

### Statistical analyses

All analyses were performed using the Statistical Package for the Social Sciences, Version 22 (SPSS, Chicago, IL, USA). Demographic variables were compared using one-way analysis of variance, Student's *t*-test, and the Chi-square test, as appropriate. Besides analysis of total participants, we also performed multiple comparison analysis between three groups (CDR 0, 1, and 1+). Because of the relatively small number of subjects with a history of depression, we used nonparametric statistics (Mann–Whitney U test) and Fisher's exact test for such comparisons.

## Results

### Demographic data

Of 181 subjects, 66 were normal (CDR = 0), 88 had MCI (CDR = 0.5), and 27 had dementia (CDR = 1 or above) (Table 1). Nine of the 181 subjects (4.9%) had a history of depressive episodes. In the three groups (dementia, MCI, and normal), 51.9, 60.2, and 53.0% were women, respectively. Comparison of the three groups showed no significant difference (*p* = 0.59). Age was significantly higher in the dementia group (84.0 vs. 80.9 vs. 79.2, *p* < 0.001). Years of education were lower in the MCI and dementia groups than in the normal group (8.8 vs. 9.3 vs. 9.9, *p* = 0.02). MMSE was lower in the MCI and dementia groups than in the normal group (17.4 vs. 22.9 vs. 24.5, *p* < 0.001). GDS was higher but not significant in the MCI and dementia groups than in the normal group (5.4 vs. 4.4 vs. 3.6, *p* = 0.18). A significantly higher proportion of the MCI and dementia groups had a history of depressive episodes (11.1% vs. 5.7% vs. 1.5%, *p* = 0.049). More atrophy of the medial temporal lobe was noted in the MCI and dementia groups (5.8 vs. 4.0 vs. 3.1, *p* < 0.001). Higher scores were obtained for CHIPS-rated WMHs in the MCI and dementia groups (32.6 vs. 20.4 vs. 17.7, *p* = 0.001). Higher scores were obtained for PVH-rated WMHs in the MCI and dementia groups (5.5 vs. 4.4 vs. 4.0, *p* = 0.006). Higher scores were obtained for DWMH-rated WMHs in the MCI and dementia groups (6.2 vs. 5.2 vs. 4.7, *p* = 0.03).

**Table 1:** Demographic data.

	**CDR = 0 *n* = 66 (36.5%)**	**CDR = 0.5 *n* = 88 (48.6%)**	**CDR = 1+ *N* = 27 (14.9%)**	***p*-value**	**Group difference**
Female (*n*, %)	35 (53.0%)	53 (60.2%)	14 (51.9%)	0.59	
Age	79.2 (4.2)	80.9 (3.8)	84.0 (5.0)	<0.001^*^	^abc^
Education	9.9 (2.2)	9.3 (1.6)	8.8 (1.7)	0.02^*^	^c^
MMSE	24.5 (3.3)	22.9 (2.9)	17.4 (5.2)	<0.001^*^	^abc^
GDS	3.6 (3.1)	4.4 (2.7)	5.4 (2.7)	0.18	^c^
CIDI-positive (*n*, %)	1 (1.5%)	5 (5.7%)	3 (11.1%)	0.049^*^	
MTA	3.1 (1.9)	4.0 (2.0)	5.8 (1.9)	<0.001^*^	^abc^
CHIPS	17.7 (16.5)	20.4 (16.2)	32.6 (20.6)	0.001^*^	^bc^
PVH	4.0 (1.8)	4.4 (2.0)	5.5 (2.1)	0.006^*^	^c^
DWMH	4.7 (2.6)	5.2 (2.2)	6.2 (2.2)	0.03^*^	^c^

*CDR, clinical dementia rating; MMSE, Mini–Mental State Examination; GDS, geriatric depression scale; CIDI, Composite International Diagnostic Interview; MTA, medial temporal atrophy; CHIPS, cholinergic pathways hyperintensities scale; PVH, periventricular white matter hyperintensity; DWMH, deep white matter hyperintensity. Chi-square test was used for comparison of gender and CIDI-positive rate; one-way analysis of variance was used for comparison of other measures*.

* *p < 0.05*.

a *Group differences: CDR = 0 vs. CDR = 0.5 (p < 0.05, adjusted for multiple comparisons)*.

b *Group differences: CDR = 0.5 vs. CDR = 1+ (p < 0.05, adjusted for multiple comparisons)*.

c *Group differences: CDR = 0 vs. CDR = 1+ (p < 0.05, adjusted for multiple comparisons)*.

### Comparison of subjects with and without a history of depression

**Figure 3:**
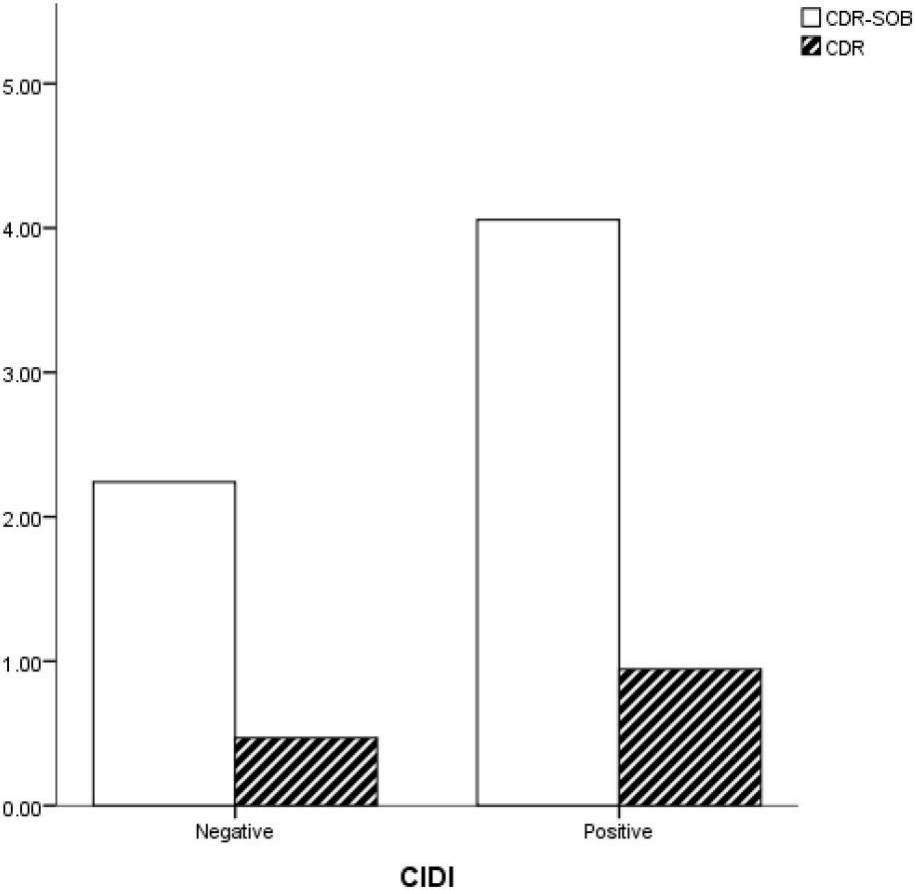
Subjects with a history of depression (CIDI-positive) showed higher CDR and CDR-SOB scores than did those without it.

Among our subjects, a lower proportion of women had a history of depression (22.2% vs. 58.1%, *p* = 0.043). No differences were observed in age, education, MMSE, and GDS between the depressed and nondepressed groups (Table 2). CDR was significantly higher in subjects with a history of depression (0.9 vs. 0.4, *p* = 0.046) (Figure 3). A smaller hippocampus was not found in these subjects (4.0 vs. 3.9, *p* = 0.898). No significant differences were observed in WMHs rated using CHIPs, PVH, or DWMH.

**Table 2:** Comparison of subjects with and without a history of depression.

	**With (CIDI-positive) *n* = 9**	**Without (CIDI-negative) *n* = 172**	***p*-value**
Female (*n*, %)	2 (22.2%)	100 (58.1%)	0.043^*^
Age	80.5 (3.7)	80.8 (4.4)	0.854
Education	10.0 (2.3)	9.4 (1.9)	0.519
MMSE	22.3 (5.4)	22.7 (4.1)	0.843
GDS	6.0 (2.3)	4.2 (2.9)	0.054
CDR	0.9 (0.9)	0.4 (0.5)	0.046^*^
CDR-SOB	4.0 (5.2)	2.2 (3.1)	0.105
MTA	4.0 (2.0)	3.9 (2.1)	0.898
CHIPS	22.4 (20.1)	21.1 (17.6)	0.858
PVH	4.7 (2.5)	4.4 (1.9)	0.744
DWMH	5.3 (2.3)	5.1 (2.4)	0.864

*CDR, clinical dementia rating; CDR-SOB, sum of boxes of clinical dementia rating scale; MMSE, Mini–Mental State Examination; GDS, geriatric depression scale; CIDI, Composite International Diagnostic Interview; MTA, medial temporal atrophy; CHIPS, cholinergic pathways hyperintensities scale; PVH, periventricular white matter hyperintensity; DWMH, deep white matter hyperintensity. Chi-square test was used for comparison of gender and CIDI-positive rate; nonparametric test (Mann–Whitney U-test) was used for comparison of other measures*.

* *p < 0.05*.

### Comparison of subjects in and not in a depressive state

**Figure 4:**
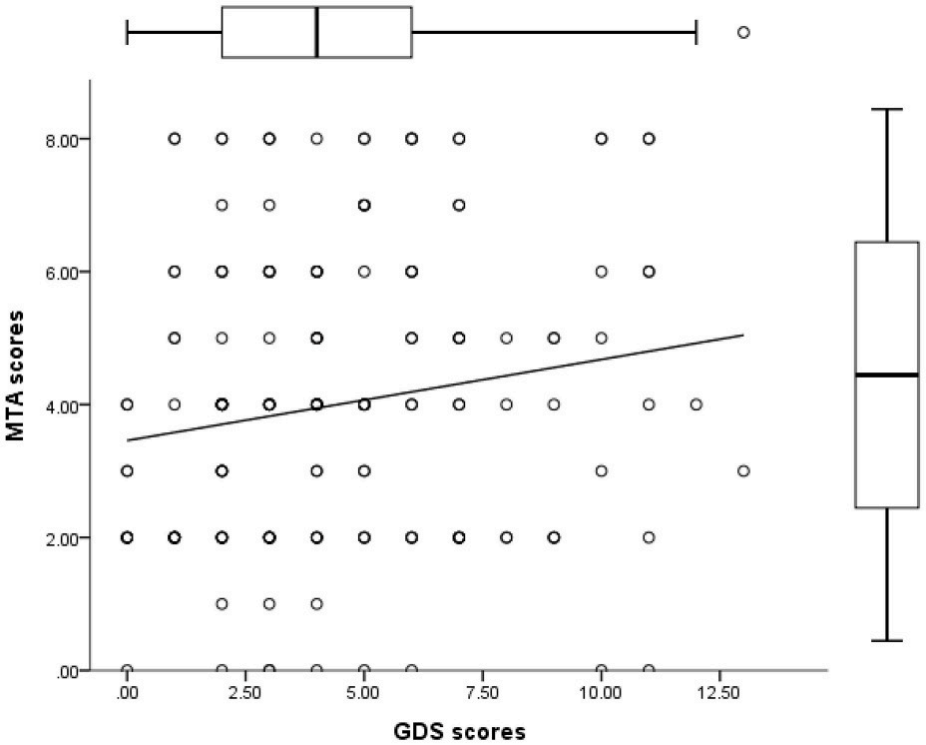
Scatterplot showing that MTA scores were positively correlated with GDS scores (*r* = 0.193, *p* = 0.009).

Of 181 subjects, 72 (39.7%) were defined as being in a depressive state (GDS ≥ 5). The proportion of women was not different between the depressed and nondepressed group (54.2% vs. 57.8%, *p* = 0.649). No differences were observed in age and education between the groups. Subjects with depression exhibited lower MMSE scores (21.6 vs. 23.3, *p* = 0.008) and higher CDR scores (0.6 vs. 0.3, *p* = 0.004), and a higher proportion of subjects had a history of depression (9.7% vs. 1.8%, *p* = 0.031). A higher proportion of dementia cases were also noted in the depressed group (22.2% vs. 10%). Subjects in a depressive state exhibited more atrophy of the medial temporal lobe (4.4 vs. 3.7, *p* = 0.036). Correlation analysis revealed that GDS scores were positively correlated with MTA scores (Figure 4). Higher scores were obtained for WMH measured using different visual rating scales, namely CHIPS, PVH, and DWMH, in depressive subjects, but these scores were not statistically significant (Table 3).

**Table 3:** Comparison of subjects with and without late-life depression.

	**Depressed(GDS ≥ 5)*n* = 72**	**Nondepressed (GDS ≤ 4) *n* = 109**	***p*-value**
Female (*n*, %)	39 (54.2%)	63 (57.8%)	0.649
Age	81.4 (4.3)	80.3 (4.4)	0.123
Education	9.4 (1.7)	9.5 (2.0)	0.616
MMSE	21.6 (4.8)	23.3 (3.5)	0.008^*^
CDR score	0.6 (0.6)	0.3 (0.5)	0.004^*^
0	17 (23.6%)	49 (45%)	0.029^*^
0.5	39 (54.2%)	49 (45%)	
1+	16 (22.2%)	11 (10%)	
CIDI-positive (*n*, %)	7 (9.7%)	2 (1.8%)	0.031^*^
MTA	4.4 (2.3)	3.7 (2.0)	0.036^*^
CHIPS	23.5 (19.0)	19.6 (16.6)	0.148
PVH	4.7 (2.1)	4.3 (1.9)	0.196
DWMH	5.4 (2.2)	5.0 (2.5)	0.260

*CDR, clinical dementia rating; MMSE, Mini–Mental State Examination; GDS, geriatric depression scale; CIDI, Composite International Diagnostic Interview; MTA, medial temporal atrophy; CHIPS, cholinergic pathways hyperintensities scale; PVH, periventricular white matter hyperintensity; DWMH, deep white matter hyperintensity. Chi-square test was used for comparison of gender and CIDI-positive rate; Student's t-test was used for comparison of other measures*.

* *p < 0.05*.

## Discussion

### Summary of results

The prevalence of dementia in our study population was 14.9%. This finding is consistent with that of a previous study in the old-old population in Japan (Meguro et al., 2012). In our population, 4.9% had a history of depression, and 39.7% had depressive symptoms (based on screening results involving the GDS). Direct comparison of subjects with and without a history of depression revealed that more men had a history of depression, and subjects with a history of depression had a worse clinical dementia state (Table 2). Subjects with depressive symptoms had lower MMSE scores, a worse clinical dementia state, and a smaller hippocampus (Table 3).

### Limitations

In this study, the number of cases was relatively small; thus, some positive results may have been overlooked because of a lack of statistical power. Because this was a community-based cross-sectional study, we did not further diagnose dementia subtypes in our patients. In the future, a longitudinal follow-up of these subjects may provide more information. Another limitation is that we did not record the age of onset for the depressive episodes. Early- and late-onset depressive episodes may have different implications for our findings.

### Lifetime history of depression in old-old population

Our study findings suggested that a lifetime history of depression is associated with a worse clinical dementia state in the old-old population. Eight of nine (88.9%) subjects with a history of depression were clinically defined as having MCI or dementia (Table 4). The lower prevalence of a history of depression compared with that in Western countries and the higher prevalence of a history of depression in men are both characteristic features of Japan (Ishikawa et al., 2016). Most previous studies have linked the history of depression to late-life vascular dementia (Hébert et al., 2000; Brunnström et al., 2013) or white matter changes (Duffy et al., 2014). However, in this study, more WMH was not detected in any subjects with a history of depression. Moreover, a smaller hippocampus was not found in any subject with a history of depression. This finding is similar to that of a previous study of Alzheimer disease (Brunnström et al., 2013). Based on our study findings, a smaller hippocampus and white matter changes may not be the common origins of lifetime depressive episodes and dementia. Although it is possible that our sample size is too small to demonstrate statistical significance, other causes might still explain both conditions.

**Table 4:** Clinical data of nine subjects with a history of depressive episodes.

**Case**	**Age**	**Gender**	**CDR**	**GDS**	**MDE**	**MMSE**
1	78	Male	0.5	2	Negative	26
2	87	Male	3	6	Positive	16
3	79	Female	0.5	5	Positive	28
4	80	Male	0.5	6	Negative	27
5	74	Male	0	4	Positive	26
6	85	Male	1	9	Positive	20
7	80	Male	2	8	Negative	12
8	81	Female	0.5	5	Negative	22
9	81	Male	0.5	9	Negative	24

*CDR, clinical dementia rating; MMSE, Mini–Mental State Examination; GDS, geriatric depression scale; MDE, major depressive episode*.

### Late-life depression and dementia

Another major point of this study is that the findings increase understanding of the relationship between late-life depression and dementia. Subjects with senile depression exhibited poor cognitive performance and more atrophy of the medial temporal lobe than did those without senile depression. The relationship between chronic depression and a smaller hippocampus has long been recognized. A previous longitudinal study even proved that late-life depression leads to hippocampal decline but not vice versa (den Heijer et al., 2011). Our target population was older than those of previous studies. Thus, we could comprehensively observe the consequences of senile depression. Furthermore, in our target population, subjects with depression had a higher prevalence of dementia and lower cognitive performance than did those without depression.

By contrast, vascular risk factors or widespread WMH changes have always been assumed to be among the possible causes of late-life depression. Most studies have reported more WMHs in people with late-life depression (Fujishima et al., 2014). In our study, although subjects with depression showed higher WMH scores (rated using CHIPS, PVH, and DWMH), the findings were not statistically significant (Table 3). However, this result might be because our subjects represent the old-old population, and white matter changes are also a part of the aging process (Gunning-Dixon et al., 2009). We might have needed more cases to reach statistical significance. Most recent studies support the notion that senile depression is a prodromal symptom of vascular dementia or all-cause dementia. Based on our findings, depression in the old-old population is related to lower cognitive performance and, potentially, future dementia development. However, to establish such a temporal relationship, a longitudinal study should be conducted in the future.

In conclusion, we found lifetime history of depression do have some effects on dementia onset in old-old population. Participants with history of depressive episodes have higher CDR scores. However, we didn't find them with more atrophy medial temporal lobe or more WMHs. In old-old population, lifetime depressive episodes may have significant effects on their clinical condition without structural changes. Previous reports have revealed more neuropathological changes in AD patients with lifetime history of major depression (Rapp et al., 2006). Our reports further support the importance of lifetime history of depression in old-old population. On the other hand, depressive state in this group of people related to smaller hippocampus. However, we didn't find depressive state significantly related to more WMHs.

## Author contributions

YL: analyzing the data and writing the manuscript KM: design the study and collecting and analyzing the data KN, KA, MN, TS: collecting the data SN, MM, NK: discussion

### Conflict of interest statement

The authors declare that the research was conducted in the absence of any commercial or financial relationships that could be construed as a potential conflict of interest.
